# Overexpression of recombinant HIV-1 Subtype C Tat and Nef in a Salmonella vaccine vector

**DOI:** 10.11604/pamj.2013.16.19.2759

**Published:** 2013-09-17

**Authors:** Nyasha Chin’ombe, Maribanyana Lebeko, Mankgopo Kgatle

**Affiliations:** 1 Division of Medical Virology, Faculty of Health Sciences, University of Cape Town, Observatory 7925, Cape Town, South Africa; 2 Department of Medical Microbiology, College of Health Sciences, University of Zimbabwe, P O Box A178 Avondale, Harare, Zimbabwe

**Keywords:** Overexpression, regulatory proteins, HIV, viral replication, recombinant, Salmonella

## Abstract

Tat and Nef are very important regulatory proteins of HIV-1. They enhance viral replication and down-regulate expression of MHC Class I molecules, respectively. The antigens are now considered to be targets for HIV vaccine development. The expression of Tat and Nef in *Salmonella* vaccines has not previously been investigated. In this study, HIV-1 Subtype C tat and nef genes were cloned into an expression plasmid and their expression investigated in *Salmonella*. Very high-level expression of the two HIV-1 antigens was demonstrated in the recombinant *Salmonella*. The antigens were also successfully purified in bulk from the bacterium.*Salmonella* can therefore potentially be used to overexpress HIV-1 antigens and used as a possible delivery system in HIV-1 vaccine development.

## Introduction

HIV-1 Tat (transactivator) and Nef (negative factor) are important regulatory viral proteins [1]. Tat enhances viral replication and Nef down-regulates major histocompatibility complex (MHC) Class I molecule expression during HIV-1 infection [2]. The two antigens are expressed early in infection and are therefore potential targets for HIV-1 vaccine development [3]. Although Tat and Nef have previously been expressed in *E. coli*, their expression in *Salmonella* vaccine vectors has not previously been investigated widely. Live *Salmonella* vaccines expressing such foreign antigens have a great potential to be used as mucosal vaccines for HIV-1. They are capable of eliciting mucosal and systemic immune responses after oral administration [4]. They are also relatively easy and cheap to produce for mass vaccinations. Our research group previously developed a recombinant *Salmonella* expression plasmid in which green fluorescent protein (gfp) and HIV-1 Subtype C gag genes were cloned and expressed at very high levels [5, 6]. We further demonstrated that oral vaccination of mice with the recombinant *Salmonella* expressing the two antigens could induce systemic antigen-specific immune responses [5, 6]. In this study, we cloned HIV-1 Subtype C tat and nef genes into the backbone of the same expression plasmid and further investigated the expression of the antigens by the recombinant *Salmonella* vaccine.

## Methods

The *tat* and *nef* genes (from primary HIV-1 Subtype C isolates, Du422 and Du151, respectively), codon-optimized for expression in *Salmonella* bacterium were cloned into the backbone of a prokaryotic expression plasmid previously reported by our group [5, 6]. A his-tag DNA sequence was also cloned in-frame between each of the genes and the beta-galactosidase alpha-gene (lacZ-alpha). The generated plasmids, pGEM + hisTat and pGEM + hisNef were used to transform E. coli and *Salmonella enterica* serovar Typhimurium (aroC mutant) as previously described [5, 6]. The IPTG-induced expression of the Tat and Nef antigens in *E. coli* was evaluated by SDS-PAGE as previously described [5, 6]. The constitutive expression of the antigens by the recombinant *Salmonella* vaccine vector was also evaluated by SDS-PAGE as previously described [5, 6]. Purification of Tat and Nef from the recombinant *Salmonella* was performed under both denaturing and native conditions using the QIA-Express Fast Start Ni + NTA kit (QIAGEN, Germany) according to manufacturer's instructions. The bacterial protein from the different fractions (total bacterial cell lysate before purification, flow-through, first wash, second Wash and protein elutions fractions) was evaluated by SDS-PAGE. The SDS-PAGE was stained with Coomassie Blue dye.

## Results

**Figure 1:**
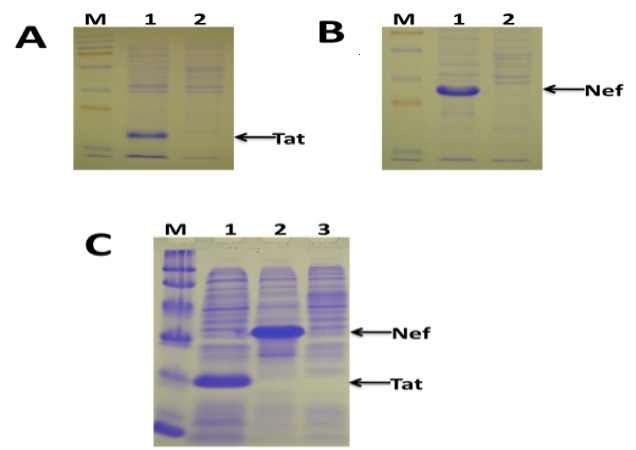
Expression of HIV-1 Subtype C Tat and Nef in recombinant E. coli and Salmonella bacteria. (A) Coomassie-stained SDS-PAGE showing Tat expression by recombinant E. coli. Lane M: Marker, Lane 1: E. coli expressing Tat, Lane 2: E. coli carrying an empty plasmid (negative control). The position of Tat protein band is indicated by an arrow. (B) Coomassie-stained SDS-PAGE showing Nef expression by recombinant E. coli. Lane M: Marker, Lane 1: E. coli expressing Nef, Lane 2: E. coli carrying an empty plasmid (negative control). The position of Nef protein band is indicated by an arrow. (C) Coomassie-stained SDS-PAGE showing Tat and Nef expression by recombinant Salmonella. Lane M: Marker, Lane 1: Salmonella expressing Tat, Lane 2: Salmonella expressing Nef, Lane 3: Salmonella carrying an empty plasmid (negative control). The positions of Tat and Nef protein bands are indicated by arrows.

**Figure 2:**
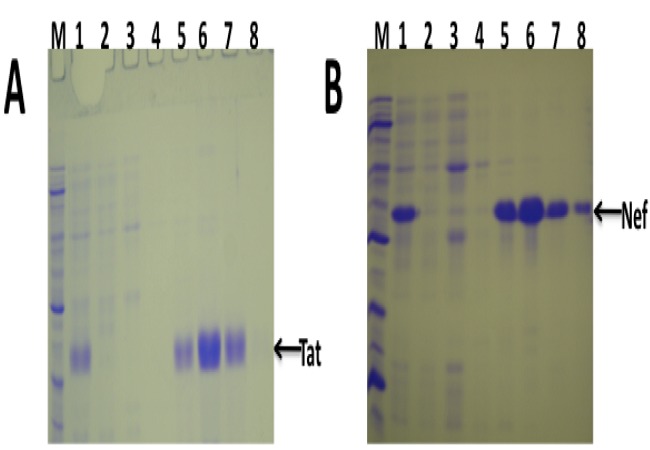
Purification of HIV-1 Subtype C Tat and Nef from recombinant Salmonella under denaturing conditions (A) Coomassie-stained SDS-PAGE showing the purification of Tat from recombinant Salmonella. Lane M: Marker, Lane 1: Total bacterial cell lysate, Lane 2: Flow-through, Lane 3: First wash, Lane 4: Second wash, Lanes 5-8: Tat elution fractions. The position of Tat protein band is indicated by an arrow. (B) Coomassie-stained SDS-PAGE showing the purification of Nef from recombinant Salmonella. Lane M: Marker, Lane 1: Total bacterial cell lysate, Lane 2: Flow-through, Lane 3: First wash, Lane 4: Second wash, Lanes 5-8: Nef elution fractions. The position of Nef protein band is indicated by an arrow.

HIV-1 *tat* and *nef* genes were successfully cloned into a prokaryotic expression plasmid previously developed by our research group. Two recombinant plasmids, pGEM + hisTat and pGEM + hisNef were successfully generated. Initial evaluation of Tat and Nef expression in *E. coli* showed very high expression of the antigens (Figure 1A and B). The Tat and Nef protein bands were visible on Coomassie-stained SDS-PAGE gels (Figure 1A, B). The specificity of the bands was confirmed by Western blotting (results not shown). The expression of the two foreign proteins was also very high in recombinant *Salmonella* (Figure 1). Both HIV-1 Tat and Nef were successfully purified in bulk from the recombinant *Salmonella* bacteria under denaturing conditions (Figure 2A, B). The purified antigens were of high purity (Figure 2A, B, Lanes 5-8). Under native conditions, only Nef, but not Tat could be purified (results not shown). The purified antigens were confirmed by Western blotting (results not shown).

## Discussion

One of the greatest challenges with the development of recombinant *Salmonella* vaccine vectors is to achieve high-level expression of the foreign antigens for successful delivery to the immune system. In our previous study, we successfully developed a *Salmonella*expression plasmid to use for the expression of foreign antigens (GFP and HIV-1 Gag) in *Salmonella* vaccine vectors [5, 6]. Immunization of mice with the recombinant *Salmonella* expressing GFP or Gag induced specific immune responses [5, 6]. In the current study, we used the same expression system to express HIV-1 Tat and Nef in *Salmonella* vaccine vector. The expression of the two foreign proteins was extremely high in both *E. coli* and *Salmonella* bacteria. To facilitate purification of Tat and Nef proteins from the bacteria, a his-Tag DNA sequence was cloned between the genes and the lacZ-alpha. The his-tag did not affect the expression of the two foreign antigens. Although both Tat and Nef were purified in bulk under denaturing conditions, only only Nef could be purified under native conditions. This suggested that Tat was expressed mainly as inclusion bodies while Nef was expressed as both soluble and insoluble protein in *Salmonella* It is a common phenomenon that some antigens may form inclusion bodies when over expressed in E. coli or *Salmonella*. Using the Wilkinson-Harrison solubility model, the probabilities of insolubility for Tat and Nef was calculated and found to be 91% and 55% respectively.

Over expression of heterologous antigens in recombinant *Salmonella* vaccine vectors without causing metabolic burden to the bacteria is a key challenge [7, 8]. In this study, we managed to express HIV-1 Tat and Nef at very high levels in a recombinant *Salmonella* vaccine vector due to a number of factors. The prokaryotic expression system which uses the transcription and translation domains in pGEM-Teasy plasmid (Promega, USA) have already previously been shown by our research group to be a good system for expression of other antigens such as GFP and HIV-1 Gag without much metabolic burden to the bacterial growth [5, 6]. Fusing the genes to the lacZ-alpha) peptide gene and use of the lac (bacterial) promoter enhanced the high expression of the antigens. The codon-optimization of the tat and nef genes also improved the high-level expression of the Tat and Nef proteins in both *Salmonella* and *E. coli* bacteria [11]. It is anticipated that the *Salmonella* expressing Tat and Nef may induce both B- and T- cell responses if used as oral vaccines in animal studies. We have already previously shown that recombinant *Salmonella* expressing GFP (mainly soluble) elicited humoral and CD8+ T cell responses while Gag which was expressed mainly as inclusion bodies elicited humoral and CD4+ T cell responses [5, 6]. The purified HIV-1 antigens can potentially be used as reagents in immunoassays for the detection of HIV-1 antibodies in clinical human specimens or in animals vaccinated with HIV-1 Tat and Nef antigens. The antigens can also be used to develop protein-based subunit vaccines for induction of antibody responses against HIV-1 infection. The recombinant *Salmonella*expressing HIV-1 Tat and Nef developed during this study can potentially be used as candidate HIV vaccine.

## Conclusion

The HIV-1 antigens, Tat and Nef were over-expressed in recombinant *Salmonella*vaccine. All the antigens were also purified in bulk. Therefore recombinant *Salmonella* is be good bacterial system for expressing foreign proteins at high levels. Such a system can also be used as a potential vaccine vector for HIV-1 antigens.
